# Occurrence and Role of Selected RNA-Viruses as Potential Causative Agents of Watery Droppings in Pigeons

**DOI:** 10.3390/pathogens9121025

**Published:** 2020-12-06

**Authors:** Ewa Łukaszuk, Tomasz Stenzel

**Affiliations:** Department of Poultry Diseases, Faculty of Veterinary Medicine, University of Warmia and Mazury in Olsztyn, ul. Oczapowskiego 13, 10-719 Olsztyn, Poland; tomasz.stenzel@uwm.edu.pl

**Keywords:** astrovirus, avulavirus, coronavirus, diarrhea, picornavirus, pigeons, rotavirus, watery droppings

## Abstract

The diseases with watery droppings (diarrhea and/or polyuria) can be considered some of the most severe health problems in domestic pigeons of various ages. Although they do not always lead to bird death, they can contribute to poor weight gains and hindered development of young pigeons and, potentially, to poor racing results in sports birds. The gastrointestinal tract disorders of pigeons may be of various etiology, but some of the causative agents are viral infections. This review article provides information collected from scientific reports on RNA-viruses belonging to the *Astroviridae*, *Picornaviridae*, and *Coronaviridae* families; the *Avulavirinae* subfamily; and the *Rotavirus* genus that might be implicated in such health problems. It presents a brief characterization, and possible interspecies transmission of these viruses. We believe that this review article will help clinical signs of infection, isolation methods, occurrence in pigeons and poultry, systemize and summarize knowledge on pigeon enteropathogenic viruses and raise awareness of the importance of disease control in pigeons.

## 1. Introduction

**Figure 1:**
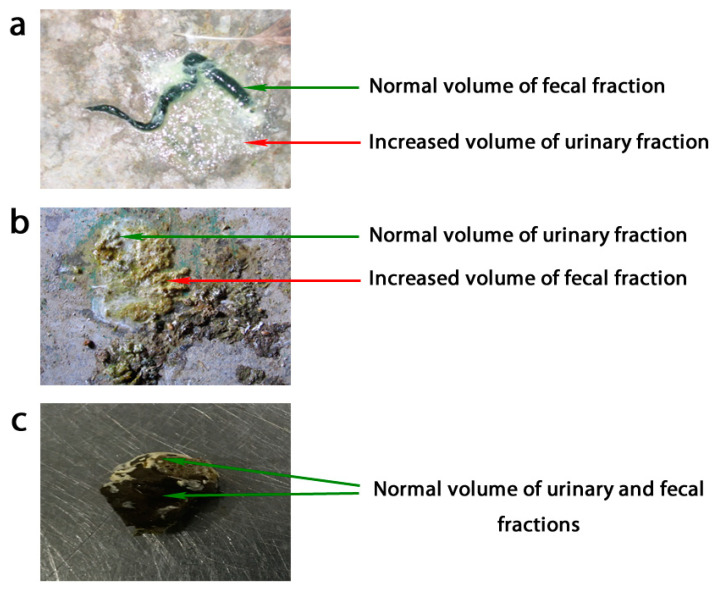
Different types of watery droppings excreted by pigeons: (**a**) watery droppings with a higher volume of the urinary fraction (polyuria); (**b**) watery droppings with a normal volume of the urinary fraction (diarrhea); (**c**) the normal droppings of a healthy pigeon.

*Columbidae* is a family consisting of more than 300 species of pigeons and doves occurring worldwide [1]. Many of them are considered synanthropic or are reared by man. Although keeping racing/homing or fancy pigeons, being breeds of domestic pigeon (*Columba livia domestica*), is fairly popular all over the world, and even though pigeons are regarded as poultry in some countries, there is still not enough research available to fully understand the epidemiology and course of diseases afflicting species from the *Columbidae* family [2]. The importance of expanding the knowledge on pigeon diseases is even greater when taking the possible consequences of contact between pigeons and commercial poultry into account. Either wild or domestic pigeons could be considered viral hosts which possibly enables evolution and recombination, leading to the development of new viral variants of different levels of pathogenicity [3]. Coronaviruses can be considered a fine example in this case due to the notable proneness to mutations [4,5]. Pigeons are often recognized as the reservoirs of multiple pathogens, which poultry and other reared animals are susceptible to. The three main reasons why pigeons pose a particular threat to other species could be recognized as: (1) the poorly described and often lacking obvious clinical signs course of infections, which impedes the effective prevention and treatment; (2) the possibility of spreading the pathogens on large distances by pigeons flying freely; and (3) relatively frequent contact between different bird species reared in households or at live bird markets [6]. In this paper, the emphasis is put on RNA-viruses from the *Astroviridae*, *Picornaviridae*, and *Coronaviridae* families; the *Avulavirinae* subfamily; and the *Rotavirus* genus, which are prevalent disease factors in various domestic animals, including poultry [7,8,9,10,11,12,13,14,15,16,17,18,19,20,21,22,23,24]. Those viruses are also isolated from domestic pigeons, but it remains unknown if the infections they cause lead to diseases of the digestive and/or the excretory tract. Such infections could result in watery droppings. This term describes both diarrhea and/or polyuria, which are pathological conditions involving an increased liquid volume (Figure 1) in avian droppings, often misdiagnosed by veterinary practitioners [25]. The diseases of domestic pigeons with watery droppings pose a severe health problem in this bird species and lead mainly to the poor development of young birds and low racing performance in sports pigeons. The purpose of this review article is to summarize the current knowledge on certain viruses occurring in the digestive/excretory tract of pigeons in the context of their putative role in pigeon enteropathies or other health disorders. The article also aims to draw attention to the potential transmission of selected enteropathogenic viruses from pigeons to commercial poultry.

## 2. Astroviruses

Avian astroviruses (Avastroviruses) are members of the *Astroviridae* family. They are small (28–30 nm), non-enveloped, positive-sense, and single-stranded RNA viruses of a star-like morphology [26]. According to the current classification proposed by the International Committee on Taxonomy of Viruses, the *Avastrovirus* genus includes chicken astrovirus (CAstV), duck astrovirus (DAstV), turkey astrovirus 1 (TAstV-1), guineafowl astrovirus (GFAstV), and goose astrovirus (GoAstV) belonging to Avastrovirus 1 species; avian nephritis virus 1 and 2 (ANV-1 and ANV-2) belonging to Avastrovirus 2 species; and turkey astrovirus 1 and 2 (TAstV-2 and TAstV-3), belonging to Avastrovirus 3 species. Our knowledge about avastroviruses is still being extended owing to in-depth investigations on astroviruses in birds conducted in the last few years. Despite that, information about host range, pathogenicity, and prevalence in wild birds is still scarce [27,28,29,30]. Astroviruses have been recognized as causative agents of the gastrointestinal disease in poultry for over 30 years and are commonly identified both in birds experiencing enteritis as well as in clinically healthy ones. The most evident signs of astrovirus infection include watery droppings and growth retardation resulting in flock unevenness (Table 1). Astroviruses are commonly associated with conditions that affect mostly young birds at the intensive growth stage, usually under 7 weeks of age in turkeys and 3 weeks in chickens. They include, e.g., TAstV being an agent in poult enteritis complex (PEC) and light turkey syndrome (LTS); CAstV as one of the causes of the runting-stunting syndrome (RSS) in chickens; and ANV causing interstitial nephritis in chickens [7,31,32,33,34,35,36].

Despite the wide distribution of astroviruses in poultry, not many reports of their detection in pigeons have been published to date. ANV and CAstV are mostly associated with diseases in chickens, manifesting with watery droppings. Zhao et al. were the first to detect these members of the *Avastrovirus* genus in pigeons in 2010, using the reverse transcription polymerase chain reaction (RT-PCR) (Table 2). Fecal samples tested for astroviruses were collected from diarrheic pigeons under 2 weeks of age during an outbreak of gastrointestinal illness in a population of Shanghai pigeons (*Columba livia*); 89% of the samples (40 out of 45) were positive for ANV and 4% (2 out of 45 samples) for CAstV, while one sample indicated infection with both ANV and CAstV [2]. Basic local alignment search tool (BLAST) analyses revealed the closest relation of the pigeon CAstV-like strain with the CAstV detected in chickens by Smyth et al. [47]. Phylogenetic analysis based on the partial polymerase gene sequence and full-length capsid protein gene from known avian astrovirus sequences revealed the close genetic relationship between detected pigeon ANV and chicken ANV. The ANV strains found in pigeons showed 83–99% identity in a 208-nucleotide region of ORF1b with the multiple reference chicken ANV strains used in the analysis, and the highest identity levels (96–97%) were shared by pigeon ANV and Chinese ANV strain Sichuan54. The results of that study suggest that the cross-infection between pigeons and chickens is indeed likely [2].

Another report of avastroviruses in pigeons involves novel viruses, other than those previously isolated from poultry. Feral pigeon astrovirus (accession numbers FR727146-FR727148) and Wood pigeon astrovirus (accession number FR727149) have been detected and characterized by Kofstad and Jonassen in cloacal and tracheal swabs sampled in Oslo from feral pigeons (*Columba livia f. urbana*) and wood pigeons (*Columba palumbus*) of unknown health status. The detection method was RT-PCR used on the stem-loop s2m part of the genome. The researchers also performed phylogenetic analyses of the partial ORF1b and complete ORF2 sequences. They revealed that the novel species had the closest similarity to ANV-1, showing 42–49% identity at the amino acid sequences encoded by ORF1b and ORF2, despite being highly divergent from it [37]. 

To date, those are the only findings of avastroviruses in pigeons, and also no reports have been published on the detection of the aforementioned pigeon astroviruses in poultry. Neither of the aforementioned research groups detected any correlation between astrovirus presence and illness development. Because the pathogenesis of the disease in pigeons is poorly understood, and astroviruses are relatively often isolated from poultry not showing any clinical signs, it remains to be determined if the astrovirus might be the direct factor causing diarrhea in pigeons (Table 1). All available data concerning pigeon astroviruses has been derived from research exploiting molecular methods. Hence, novel laboratory methods for the isolation and cultivation of these viruses should be developed to enable proving their pathogenicity. These methods could also be useful in future research aimed at identifying the genuine role of pigeon astroviruses in enteropathies and the likelihood of the cross-species contamination by challenging pigeons and poultry with their isolates.

## 3. Picornaviruses

The *Picornaviridae* is a family of small, icosahedral viruses with single-stranded, highly diverse positive-sense RNA genomes and three capsid proteins with b-barrel folding. The family comprises 63 genera containing 147 species, but many viruses are presently awaiting classification. Picornaviruses have been identified from various vertebrates, including many bird species, like chickens, turkeys, ducks, geese, quail, pheasants, cranes, passerine, penguins, parrots, and others. [27,51,52] Viruses from the *Avihepatovirus*, *Megrivirus*, and *Tremovirus* genera, infecting mostly poultry, have also been isolated from pigeons [38].

Avian encephalitis virus (AEV), belonging to *Tremovirus* genus, is the agent responsible for the development of neurotropic disease mostly in young chickens, although it has also been reported in turkeys, pheasants, and quails [51,53]. It is the first member of the Picornaviridae family to have been proven to infect pigeons. In 2003 AEV antigen has been found in multiple tissue samples of pigeons from a flock in Turkey exhibiting nervous signs, apathy and diarrhea. The researchers performed immunochemistry using a conjugate of chicken anti-AEV serum with flourescein isothiocyanate (FITC) to stain the tissues. The highest amounts of AEV antigen were found in the pancreas, kidneys, gastrointestinal tract, and brain. The results of this research not only reveal a disease that has not been reported in pigeons previously, but also suggest a possibility of cross-infection with AEV between chickens and pigeons [38]. Duck hepatitis A virus 1 (DHAV-1), strain FJ1220 (accession number KC904272) belonging to the *Avihepatovirus* genus, has been detected with the RT-PCR method in pigeons from a farm in China experiencing a hepatitis outbreak with high mortality. Unfortunately, there is no specific information available about the course of the disease in pigeons. However, this finding indicates that the pigeons not only can be a reservoir of the virus causing the worldwide infection of high fatality rate in ducklings, but are also susceptible to the disease [54].

Two other novel viruses belonging to the *Picornaviridae* family, i.e., Pigeon picornavirus A (PiPV-A) (accession number FR727145) and Pigeon picornavirus B (PiPV-B) (accession number FR727144), have been discovered by Kofstad and Jonassen in the research mentioned above. The screening of feral pigeons of unknown health status for s2m-harboring viruses revealed PiPV-A and PiPV-B in 30% of the tested birds. This finding might lead to a conclusion that picornaviruses are common in the feral pigeon population. These researchers have also found out that the closest relative of PiPV-B is the Duck picornavirus, now known under the name Anativirus A, sharing 52% of the amino acid sequence identity in the 3D genome region with PiPV-B. Pigeon picornaviruses have been classified as sapelo-like viruses, showing similarities in the genome sequence with viruses belonging to the *Sapelovirus* genus [37]. However, in today’s taxonomy, PiPV remains unassigned to a genus [52]. 

Another two picornaviruses, named Pigeon mesivirus 1 (accession number KC876003) and Pigeon mesivirus 2 (accession number KC811837), have been revealed with RT-PCR by Phan et al. examining wild pigeon fecal samples collected in Hong Kong and Hungary (Table 2). These researchers also characterized their two complete genomes. The two isolates were found to be related, sharing an overall 81% nucleotide similarity across their entire genomes, which suggests that this viral clade is widely distributed across the world [48]. The polyprotein BLASTx hits showed the closest identity to the turkey hepatitis virus belonging to Megrivirus C species of the *Megrivirus* genus in 85% of the picornavirus sequences [55]. Pigeon mesivirus 1 and 2 have been assigned to Megrivirus B species of the *Megrivirus* genus [27]. The analysis of the similarities of mesiviruses and other picornaviruses also showed that the mesivirus untranslated 5′mRNA region contained structures that were also seen in avian-origin picornaviruses, including Duck hepatitis A virus type 1, Quail picornavirus, and Pigeon picornavirus B, belonging to different genera [48].

Detection of two poultry-origin picornaviruses in pigeons should make us cautious about putative interspecies transmission, although the novel picornaviruses found in pigeons have not been reported in poultry to date. While picornaviruses are known infectious agents leading to enteric problems in commercial poultry, besides AEV, no picornavirus infection has been proven to cause enteric pathology in pigeons [51,53,56]. Based on the current knowledge, there is no data indicating a clear correlation between picornavirus infection of pigeons and their health status (Table 1). For this reason, further research would be needed to explore the pathogenicity of pigeon picornaviruses.

## 4. Coronaviruses

Coronaviruses, being members of the family *Coronaviridae* and subfamily *Orthocoronavirinae*, are enveloped, positive-sense, and single-stranded RNA viruses of mammals and birds. Virions are spherical, 120–160 nm, typically decorated with surface projections. In electron micrographs of spherical particles, these projections create an image reminiscent of the solar corona, which inspired the name of the family [57]. There is a high diversity of coronaviruses prevalent in birds, which seem to be excellent viral hosts enabling the evolution and dissemination of the viral factors [58]. Coronaviruses occurring in birds are mainly representatives of the *Deltacoronavirus* and *Gammacoronavirus* genera, and have been isolated in numerous bird species, including domestic ones like chickens, turkeys, ducks, geese; and wild ones like pheasant (*Phasianus colchicus*), rock pigeon (*Columba livia*), Chinese bulbul (*Pycnonotus sinensis*), red-whiskered bulbul (*Pycnonotus jocosus*), white-rumped munia (*Lonchura striata*), scaly-breasted munia (*Lonchura punctulata*), gray-backed thrush (*Turdus hortulorum*), blackbird (*Turdus merula*), Japanese white-eye (*Zosterops japonicus*), Eurasian tree sparrow (*Passer montanus*), oriental magpie-robin (*Copsychus saularis*), night heron (*Nycticorax nycticorax*), common moorhen (*Gallinula chloropus*), green-cheeked Amazon parrot (*Amazona viridigenalis*), Eclectus parrot (*Eclectus roratus*), and others [49,58,59,60,61,62,63,64]. Such a wide distribution of viruses from the Coronaviridae family among different bird species might be due to their unique RNA replication mechanism, being the cause of the high frequency of recombination and relatively high mutation rates; a combination of which allows the rapid evolution and adaptation to different hosts [4,5].

Members of the *Coronaviridae* family are often associated with respiratory tract infections. Interestingly, the infectious bronchitis virus (IBV), a known causative agent of respiratory disease in chickens, also replicates at many non-respiratory epithelial surfaces. For example, IBV may cause pathology in intestines, kidneys, or female reproductive tract [62]. Replication at enteric surfaces normally does not result in clinical disease, but in fecal excretion of the virus. The reinfection in the same bird or cloacal infection in the others can occur through the anal sucking and subsequent movements of the lower parts of the intestine [65]. Replication in the kidney leads to nephrosis/nephritis, which causes polyuria manifested as an increased liquid volume in droppings, indistinguishable from diarrhea in birds (Table 1) [40,66]. However, the pathogenic effect of IBV is not limited to galliform birds only. The isolation of IBV on chicken embryos was reported by Barr et al. in 1988. Fertile eggs were inoculated with a suspension made from tracheal and cloacal swabs sampled from racing pigeons that exhibited ruffled feathers, dyspnea, and excessive mucus at the beak. Interestingly, when the virus was experimentally inoculated into pigeons and chickens, the chickens developed respiratory disease, while pigeons remained healthy. The researchers speculated that IBV might have caused disease in the racing pigeons tested because their resistance was suppressed by the intercurrent disease [67].

Felippe et al. examined 23 tracheal swab samples from domestic chickens distributed in three regions of Brazil and six tracheal and cloacal swab samples from asymptomatic feral pigeons for IBV and other avian coronaviruses, using RT-PCR and nested polymerase chain reaction. The positive samples were inoculated into specific pathogen-free embryonated chicken eggs for virus isolation, then sequenced, and phylogenetically analyzed. One of the major revealed phylogenetic groups was found to be similar to the Massachusetts vaccine serotype used in Brazil, and the other was different from the vaccine strain, grouping with the D207 strain. Five pigeon isolates (accession numbers HM561878-HM561882) clustered with the IBV vaccine serotype showed similarity in the studied S1 genome fragment, approximating 100% to those obtained from chickens. This finding might be considered alarming because the detection of virus strains nearly identical to chicken strains in 5 out of the 6 samples implies the possible transfer of IBV between pigeons and chickens. One pigeon isolate (accession number HM561883) was similar to the Connecticut strain that is not used in Brazil as a vaccine. Its occurrence in pigeons could be explained by the use of an unauthorized vaccine in poultry. No samples collected from pigeons grouped with the D207 cluster that has been isolated only from chickens since 2007, which could suggest that this strain has not adapted to pigeons [3].

Novel coronaviruses have been isolated from pigeons in multiple further investigations. Jonassen et al. detected Pigeon coronavirus (accession number AJ871023) in cloacal swabs of 2 out of the 100 examined feral pigeons and in a tracheal swab of one pigeon, also positive from the cloacal swab sample, by using RT-PCR, confirmed by sequencing (Table 2). None of the coronavirus-infected pigeons showed any clinical signs nor significant pathological lesions at necropsy. There are also no data concerning diarrhea in coronavirus–positive pigeons. Coronavirus was detected in the liver and spleen of the bird from which the organ samples were available. Interestingly, the virus was not detected in the lungs despite its presence in the tracheal swab, leaving the question about its transmission route unanswered [49]. 

Qian et al. carried out an experiment in which the specific pathogen-free (SPF) embryonated chicken eggs were inoculated with the suspension containing a novel pigeon coronavirus strain PSH050513 (accession number DQ160004) prepared from the swollen pancreas of pigeons with an upper respiratory tract infection. After the serial passages, 6 clinically normal 30-day-old pigeons and 20 15-day-old White Leghorn SPF chickens were inoculated with the allantoic fluid harvested from the inoculated eggs. From day 20 post-inoculation, clinical signs of respiratory tract infection, depression, appetite loss, polydipsia, and ruffled feathers were observed in the infected pigeons. Distinct hemorrhage and excess mucus in the trachea, pulmonary lesions as well as swollen and hyperemic pancreas were revealed by necropsy. No clinical signs of digestive tract disorders as well as post-mortem lesions of alimentary and excretory systems were noticed in the inoculated birds (Table 1). As for the chickens, all developed signs of respiratory tract infection. Necropsy of the dead chickens revealed pancreatitis, similarly to pigeons. The molecular characterization of the isolated coronavirus revealed that the spike (S) glycoprotein gene was highly homologous (79.3–99.6%) with the S protein gene sequences of the avian infectious bronchitis virus. However, only limited identity (<37.8%) was observed with the turkey coronavirus and mammalian coronaviruses. Compared to other coronaviruses, these findings indicate greater similarity of the pigeon coronavirus tested with IBV strains, which in chickens mostly induce respiratory disease [41]. The similarity of the new strain and IBV might suggest the likely cross-infection between pigeons and poultry, although additional studies would be needed to assess this threat.

Lau et al. detected a novel pigeon coronavirus (accession number LC364344) while testing fecal samples of birds of unknown health status in Dubai with RT-PCR. Coronaviruses of falcon, houbara bustard, and quail have also been found, and complete genome sequencing revealed that 4 novel strains belonged to the same *Coronavirus* species. This finding, on the one hand, suggests that coronaviruses can spread from an infected prey to a bird of prey, what could prove the wide interspecies distribution of these viruses; however, on the other hand, the presence of viruses of prey in feces of bird of prey can result from diet contamination [5].

In a recent study, Martini et al. investigated the pathogenicity of three avian coronavirus isolates, i.e., pigeon coronavirus previously isolated by Felippe et al. (accession number HM561882) and two IBV strains isolated from chickens with respiratory signs, in one-day-old specific-pathogen-free White Leghorn chicks [3]. Intraocular and intranasal inoculation was performed and was followed by an outbreak of respiratory signs in all infected chickens. Congestion and catarrhal exudates in the trachea were observed at the necropsy, while the lesions in other tissues were mild or non-existent. Complete tracheal ciliostasis was observed over the following days of infection. Viral RNA was identified in all tissues tested, suggesting that the pigeon coronavirus strain and the chicken strain could replicate in various places in the body, although the highest viral RNA levels were detected in the digestive tract. Infection with the pigeon isolate also stimulated the highest humoral response in the chickens tested [68].

Most recently, Zhuang et al. performed the surveillance of coronavirus prevalence in poultry, pigeons, and wild birds in China using a conserved RT-PCR assay. They concluded that the coronaviruses most frequently isolated from pigeons belonged to different lineages than the coronaviruses isolated from the other bird species examined. Pigeon-dominant coronaviruses were detected in 23.14% (159/687) of the pigeon samples, at a significantly higher rate than in the other samples, suggesting that those viruses, while occurring in other bird species, mainly circulate in pigeons. These researchers also proved the high prevalence of pigeon coronaviruses in China, especially at live poultry markets, yet the pathogenicity of these viruses remains unknown [69]. 

Although most of the investigations mentioned above report respiratory diseases induced by coronaviruses, several viruses belonging to this family are responsible for causing enteropathies. In birds, it is undoubtedly the Turkey coronavirus (TCoV), causing an acute, highly infectious disease of high morbidity and varying mortality, affecting turkeys of all ages. The clinical signs of this condition include watery droppings, lack of appetite, and weight loss leading to the uneven flock growth (Table 1) [39]. This infection is worthy of mention because of its clinical picture corresponding to the health problems in pigeons described in this paper. So far, however, there is no literature data available proving TCoV occurrence and pathogenicity in pigeons.

While proving the high genetic diversity and variability of avian coronaviruses, all of the mentioned studies may also suggest a close connection between strains occurring in pigeons and poultry, which could be treated as a potential threat of the cross-infection between those two groups of birds. Wille and Holmes suggest that viruses from the *Deltacoronaviridae* and *Gammacoronaviridae* subfamilies should be included in future viral monitoring programs because cross-species transmission is a common feature of coronaviruses and, therefore, the risk of disease spreading between wild birds and poultry is very high [70]. Although coronaviruses inducing an enteropathogenic effect in poultry are known, the same has not been proven in pigeons yet. Because most of the thus-far studies concerning the coronavirus infection in pigeons have strived to search for a relationship with IBV and respiratory tract diseases, further research on coronavirus prevalence in the gastrointestinal tract of pigeons and its potential role in enteropathies would be advisable.

## 5. Rotaviruses

Viruses from the *Rotavirus* genus, belonging to the *Sedoreovirinae* subfamily and the *Reoviridae* family, are non-enveloped, double-stranded RNA viruses containing triple-layered capsid and resembling a wheel (Latin-“rota”) under a negative contrast electron microscope. To date, species designated A to I have been known, and Rotavirus J has been proposed most recently. Rotaviruses are transmitted by a fecal–oral route and cause infections of the intestinal tract in various avian and mammalian species, mostly in young individuals [71,72,73,74]. Rotaviruses of groups A, D, F, and G were detected in many bird species, such as chickens, turkeys, ducks, and wild birds [75,76,77,78,79,80]. 

Minamoto et al. were the first to isolate rotaviruses from pigeons in 1987 in Japan. Two new strains belonging to group A (RVA), designated as PO-8 and PO-13, were found by treating the fecal samples with trypsin and inoculating roller tube cultures of mammalian cells. A serological survey using hemagglutination inhibition and neutralization tests was then performed. Its results indicated that the antibodies to the pigeon rotaviruses were widely spread in both chicken and feral pigeon populations in Japan at that time [81]. Rotavirus isolated from a calf with diarrhea was identified as a strain of pigeon rotavirus PO-13 (accession number L41492) by Rohwedder and others, suggesting that rotaviruses can not only cross the species barriers in nature but can also be transmitted between different classes of vertebrates [82]. Mori et al. induced diarrhea in suckling mice by orally inoculating them with a pigeon rotavirus, PO-13, while turkey rotavirus, Ty-3, did not express any virulence even after inoculation with its maximal dose tested [83].

McCowan et al. identified a previously undescribed group A rotavirus of avian origin (accession numbers MH668302-MH668312) using next-generation sequencing and isolation in cell lines, which was also the first report of extra-intestinal rotavirus infection in an avian species. They investigated cases of vomiting and diarrhea connected with high mortality in domestic pigeons in Australia (Table 1). The histological examination revealed prominent hepatic necrosis, while non-enveloped viral particles of a wheel-like appearance typical of viruses from the *Rotavirus* genus have been found using the negative contrast electron microscopy of liver preparations [42]. Another occurrence of rotavirus A hepatic necrosis in pigeons was noted in California by Blakey et al., who examined the samples acquired from the racing pigeons and squab breeders showing symptoms similar to those described by McCowan and others [42]. They performed immunohistochemistry and negative-stain electron microscopy tests as well as subsequent phylogenetic analysis to identify the virus [84]. Moreover, several previously unknown RVA lineages closely related but not identical to an RVA variant identified in Australian cases were discovered by Rubbenstroth et al., who examined a series of outbreaks of acute disease with the clinical picture similar to the cases mentioned previously, reported in domestic and feral pigeons in Germany, Belgium, and Denmark. The sequence analysis indicated that the RVA lineages revealed have been circulating in Europe since at least 2010 (Table 2) [43]. Interestingly, while the RVA-associated disease in Europe closely matched the description of Australian pigeon RVA outbreaks, it had also considerable similarities (among others diarrhea, vomiting, crop stasis, loss of appetite and acute course of the disease) with the young pigeon disease syndrome (YPDS), a disease of still poorly understood etiology observed in European pigeon populations [85,86]. Rubbenstroth and others performed an experimental peroral inoculation of healthy juvenile homing pigeons with two genetically different RVA isolates, which led to a development of acute YPDS-like disease with 100% morbidity in all infected birds. Therefore, they proved the pigeon-associated group A rotavirus G18P (accession numbers MH568745-MH568794) to be one of the primary pathogens causing a disease corresponding with the clinical picture of YPDS [87]. 

The first group G rotavirus (RVG) in pigeons, different from the previously described chicken rotavirus has been found in 2013 by Phan et al. They genetically characterized the genome of the highly divergent rotavirus (HK18) (accession numbers KC876005-KC876015) isolated from feces of wild pigeons of unknown health status with the use of RT-PCR [48,72,75]. A genome characterization of Turkey Rotavirus G strains (Minessota-1 and Minessota-2) performed by Chen et al. showed relatively low nucleotide percentage identities (31.6–87.3%) between the pigeon and chicken RVG strains, proving high genetic diversity within the RVG group exclusive to avian species [88]. 

Summarizing the reports on rotaviruses in pigeons, we can observe that viruses from RVA group might be transferred not only between bird species, but even between different classes of vertebrates. In contrast, cross-species infection with viruses from RVG, a genetically diverse group, has not been proven. Combined with the documented impact of RVA on the occurrence of enteric diseases in pigeons, one may come to a conclusion that further research on RVA is needed to gain knowledge necessary to prevent the spread of rotavirus-induced illness in various animal species.

## 6. Viscerotropic Avulaviruses

*Avulavirinae* is a subfamily of viruses in *Paramyxoviridae*, the family of pleomorphic enveloped viruses that contain a single-stranded, non-segmented negative RNA genome [89]. Members of the subfamily, divided into three genera—*Metaavulavirus*, *Orthoavulavirus*, and *Paraavulavirus*, are collectively known as avulaviruses or avian paramyxoviruses as they primarily infect birds, including poultry and wild birds. To date, the subfamily comprises 22 species, according to the International Committee on Taxonomy of Viruses [27]. Some isolates still await classification—e.g., novel metaavulavirus circulating in doves and pigeons but avirulent for chickens, discovered by Liu et al. in samples from clinically healthy red turtle doves (*Streptopelia tranquebarica*) from Taiwan (accession number MK677430) [90].

Avian avulavirus 1 (AAvV-1), also known as avian paramyxovirus 1 (APMV-1) or Newcastle disease virus (NDV), belonging to the *Orthoavulavirus* genus, is the most characterized species of the avulaviruses because of the severity of the disease it causes in poultry, even though it infects a significant number of bird species, including domestic and wild ones [91]. The clinical picture of the disease depends on the host species, host immunity, and strain virulence [24,92]. Although the clinical picture may not be obvious and some viruses are not easily signed to a specific pathotype, NDV isolates have been classified into five pathotypes, based on the disease observed in chickens under laboratory conditions, namely: (1) viscerotropic velogenic-strains that cause a highly virulent form of the disease with hemorrhagic lesions in the intestinal tract; (2) neurotropic velogenic-strains that cause high mortality following respiratory and nervous signs; (3) mesogenic-strains that cause respiratory and sometimes nervous signs with low mortality; (4) lentogenic-strains that cause mild or unapparent respiratory infections; and (5) asymptomatic enteric-strains that cause unapparent enteric infections [93,94]. 

Avulaviruses have been isolated from several species from the *Columbidae* family, including among others: domestic pigeon (*Columba livia domestica*), rock pigeon (*Columba livia*), mourning dove (*Zenaida macroura*), and collared dove (*Streptopelia decaocto*) [95,96,97]. However, a lack of unambiguous clinical signs and pathological lesions may leave Newcastle disease unrecognized in the non-gallinaceous birds [98]. Erickson et al. observed submucosal hemorrhages of the gastrointestinal tract after experimentally infecting racing homer pigeons with viscerotropic velogenic AAvV-1, while lesions found in chickens are typically erosive to ulcerative [99]. Ellakany et al. proved that the shedding of the virus was higher in the oropharynx than in the cloaca, and that the clinical signs and mortality were less severe in the pigeons infected with viscerotropic velogenic AAvV-1 either intramuscularly or intranasally as opposed to the chickens being in contact with the infected pigeons [100]. The viscerotropic velogenic type of AAvV-1 was also found by Kaleta and Marschall in a pied imperial pigeon (*Ducula bicolor*) kept in a zoo in close proximity to young demoiselle cranes (*Grus virgo*), newly imported from their natural habitat (Table 2). The birds died within the span of 10 days without obvious clinical symptoms, although necropsy revealed lesions in their liver, spleen, and intestines [98]. 

An antigenic and host variant of AAvV-1 causing Newcastle disease-like infection and pathology in pigeons is called pigeon paramyxovirus type 1 (PPMV-1) (accession number AJ880277). It is speculated that PPMV-1 emerged from AAvV-1 due to multiple events of interspecies transmission, presumably chicken-to-pigeon [6]. PPMV-1 was first detected in the Middle East during the late 1970s, and the disease caused by it has now been recognized for over 30 years, continuing to circulate in racing and feral pigeon populations [24,101]. Clinical signs of PPMV-1 infection usually resemble the symptoms caused by neurotrophic velogenic NDV strains including, for example, locomotor disturbances of limbs, torticollis, and watery green diarrhea [102]. However, disease development caused by viscerotropic strains exhibiting specific affinity to the kidneys has also been reported. In this case, the most distinct sign is polyuria, while the neural symptoms appear later and only in individual birds in the flock (Table 1) [45]. The mortality of this form of the disease usually does not exceed 10%, although it may reach 30% with intercurrent bacterial or parasitic infections [103]. Nonetheless, polyuria is often wrongly recognized by veterinary practitioners as diarrhea. Because avian droppings contain both urates and intestinal digesta, both pathological states mentioned above are easily confused with each other (Figure 1) [25]. To summarize, avulavirus infection in pigeons can lead to diarrhea, but more often to polyuria. For this reason, viral kidney infections should be considered a potential cause of watery droppings.

Despite being highly adapted to pigeons, PPMV-1 is considered a threat to poultry [104]. Its spread to chickens has occurred in several countries, including Great Britain, where over 20 outbreaks were reported in 1984 in unvaccinated chickens administered feed contaminated by infected pigeons [105]. Kommers et al. proved the increased virulence of the PPMV-1 isolates tested after passage in chickens, although the only clinical symptoms observed included depression and nervous signs occurring in only some of the birds [106]. More recently, some PPMV-1 strains have been reported to be highly pathogenic for chickens after serial passages in chickens, indicating their potential to cause ND outbreaks [107,108].

Besides PPMV-1, another avulavirus serotype was found to occur in the *Columbidae* family. Avian avulavirus 7 (AAvV-7) (accession number FJ231524), assigned to the *Metaavulavirus* genus, was isolated by Alexander et al. from hunter-killed doves and classified as a new serotype based on haemagglutination inhibition (HI) and neuraminidase inhibition (NI) assays [96]. AAvV-7 has later been reported from other avian species, such as ostriches and turkeys [109,110]. In 2008, 27% of commercial chickens tested using the hemagglutination inhibition test were found to be seropositive for AAvV-7, suggesting its relatively high prevalence in the USA. However, the antibodies to AAvV-1 were detected in as many as 71% of the birds examined [111]. AAvV-7 infection has not been associated with severe disease conditions, neither in poultry nor pigeons. Also, there is no literature data concerning a correlation between AAvV-7 infection and watery droppings in pigeons.

Because cases of the spread of highly pathogenic avulaviruses from pigeons to poultry, and the other way round, have been proven, it is essential to continue the monitoring of *Avulavirus* prevalence in both poultry and wild birds. Further studies on avulaviruses circulation between pigeons and poultry could help to implement measures that can be taken to prevent or mitigate viral transmission to poultry.

## 7. Conclusions

The occurrence of watery droppings is a common and serious clinical symptom in domestic pigeons that leads to decreased racing performance and suppressed development of young birds. Viral enteropathies pose a severe problem in poultry production worldwide, but can also affect domestic pigeons. However, in the case of poultry, those diseases could be caused by mixed viral infections [12,112]. Moreover, because many enteric viruses can be detected in clinically healthy individuals, they could be a part of the normal microbiome of pigeons’ digestive tract. The summarized available research data indicates that not all viruses mentioned in this review article can cause infections with diarrhea/polyuria in pigeons. Respective knowledge gaps concern mainly pigeon astroviruses, pigeon picornaviruses, and pigeon coronaviruses. It is also noteworthy that some viruses that are not a direct causative agents of digestive tract disorders could act as interfering agents or could predispose to clinical infections, like enteropathies, by inducing immunity suppression. The best known, immunosuppressive virus in pigeons is the pigeon circovirus, which was suspected to be a causative agent of YPDS [86,113]. The current knowledge depreciates its direct role in this disease syndrome; however, little is known about the influence of PiCV-induced immunosuppression on the development of digestive tract disorders in pigeons. 

All literature data discussed in this paper reflects upon the detection of viruses using molecular diagnostic tools (e.g. RT-PCR) that allow to detect a single infection. Research based on other molecular techniques would be advisable for better understanding of the real role of the viruses mentioned in this paper. A promising alternative could be viral metagenomics (viromics), which is based on the unbiased amplification of genetic material rather than the amplification of conserved genes that are not shared by all viruses, allowing the simultaneous detection of all the members of viral communities (thus the detection of co-infections), and putatively to the discovery of new viral agents responsible for watery droppings [114]. 

The Koch’s postulates should be fulfilled to prove the role of certain infection agents in disease development. However, fulfilling all of them is impossible in the case of many viruses, because there are no methods for laboratory propagation of numerous viruses, including those described in this paper. The novel metagenomic Koch’s postulates, which focus on the identification of metagenomic traits in disease cases, could be a very interesting alternative in this case. According to Mokili et al. (2012), the metagenomic traits found in diseased individuals can be monitored in the healthy ones exposed to the suspected infectious agent [115]. Unlike the original Koch’s postulates, this novel approach requires isolating the remaining co-occurring disease candidates, but not necessarily the pathogen in the tissue culture or pure culture media. The metagenomic research not only would allow the true role of the described viruses in the enteropathies to be determined, but would also lead to the discovery of novel, undescribed pigeon viruses.

Pigeons can be a reservoir of various pathogens, often with a subclinical or atypical course of the disease, which allows them to spread between individuals, providing an opportunity for viral passages and mutations. Although intensification of the production and rise of biosecurity measures significantly reduce the chances of contact between pigeons and poultry, the potential interspecies transmission is still a threat to free-range flocks and those from amateur rearing. Extensive production systems mean that poultry is more exposed to pathogens of other animals. Moreover, an accidental contamination of, e.g., feed or bedding should also be considered even in the farms following high biosecurity standards. Considering the above conclusions, the occurrence of infectious diseases in pigeons raises concerns not only among pigeon keepers but also among breeders of poultry and other birds. For those reasons, research should be continued to find a correlation between the aforementioned viruses and disorders of digestive and excretory tracts in species from the *Columbidae* family.

## Figures and Tables

**Table 1:** The comparison of the role of selected viruses in the occurrence of diarrhea/polyuria (watery droppings) in pigeons and poultry.

Infection	Diarrhea/Polyuria Confirmed	Reference
Avian nephritis virus (ANV)	Yes, >2-week-old pigeons Yes, 3- to 7-day-old chickens	Zhao et al., 2011 [2] Shirai et al., 1991 [32]
Chicken astrovirus (CAstV)	Yes, >2-week-old pigeons Yes, 3-week-old chickens	Zhao et al., 2011 [2] Baxendale and Mebatsion, 2004 [34]
Turkey astrovirus (TAstV)	Yes, 1- to 4-week-old turkeys	Reynolds and Saif, 1986 [31]
Pigeon picornavirus (PiPV)	No	Kofstad and Jonassen, 2011 [37]
Avian encephalitis virus (AEV)	Yes, adult pigeons	Toplu and Alcigir, 2004 [38]
Turkey coronavirus (TCoV)	Yes, turkeys of all ages	Nagaraja and Pomeroy, 1997 [39]
Infectious bronchitis virus (IBV)	Yes, 4-week-old chickens	Pohl, 1974 [40]
Pigeon coronavirus strain PSH050513	No	Qian et al., 2006 [41]
Rotavirus A (RVA)	Yes, domestic pigeons of all ages, but mainly young birds Yes, 5- to 14-day-old chickens	McCowan et al., 2018 [42] Rubbenstroth et al., 2019 [43] Otto et al., 2006 [44]
Pigeon paramyxovirus type 1 (PPMV-1)	Yes, in pigeons	Pestka et al., 2014 [45]
Avian avulavirus 1 (AAvV-1)	Yes, 3- and 6-week-old chickens Yes, 13- and 19-week-old turkeys	Alexander et al., 1998 [46]

**Table 2:** Molecular diagnostic procedure of selected viruses putatively causing digestive and/or excretory tract disorders in pigeons.

Infection	Sample Type	Method	Amplified Gene	Reference
Avian nephritis virus (ANV)	Fecal sample	Reverse transcription polymerase chain reaction (RT-PCR)	ORF2	Zhao et al., 2011 [2]
Rotavirus A (RVA)	Fecal sample, cloacal swab, tissue sample (liver, spleen, intestine, cloacal bursa, lung, kidney, heart, thymus, brain, vagal nerve)	RT-PCR	VP6, NSP4	Rubbenstroth et al., 2019 [43]
Pigeon mesivirus 1 and 2 (MeV-B1, MeV-B2)	Fecal sample, cloacal swab	RT-PCR	RNA-dependent RNA polymerase	Phan et al., 2013 [48]
Pigeon coronavirus (PCoV)	Cloacal swab, tracheal swab	RT-PCR	Replicase gene	Jonassen et al., 2005 [49]
Avian avulavirus 1 (AAvV-1)	Tracheal, oropharyngeal or cloacal swabs	Reverse transcription quantitative polymerase chain reaction with TaqMan probes (RT-TaqMan qPCR)	Matrix protein (M) gene + fusion protein (F) gene	Wise et al. 2004 [50]
